# The relationship and pathways between resting-state EEG, physical function, and cognitive function in older adults

**DOI:** 10.1186/s12877-024-05041-x

**Published:** 2024-05-27

**Authors:** Hairong Liu, Jing Wang, Xin Xin, Peng Wang, Wanting Jiang, Tao Meng

**Affiliations:** 1 Physical Education Department of Shanghai International Studies University, Shanghai, China; 2 School of Sports and Health of Shanghai Lixin University of Accounting and Finance Shanghai, Shanghai, 201620 China; 3 Shanghai University of Sport, Shanghai, China

**Keywords:** Older adults, Cognitive impairment, Physical function, Cognitive function, Electroencephalography (EEG)

## Abstract

**Objective:**

Based on resting-state electroencephalography (EEG) evidence, this study aimed to explore the relationship and pathways between EEG-mediated physical function and cognitive function in older adults with cognitive impairment.

**Methods:**

A total of 140 older adults with cognitive impairment were recruited, and data on their physical function, cognitive function, and EEG were collected. Pearson correlation analysis, one-way analysis of variance, linear regression analysis, and structural equation modeling analysis were conducted to explore the relationships and pathways among variables.

**Results:**

FP1 theta (effect size = 0.136, 95% CI: 0.025–0.251) and T4 alpha2 (effect size = 0.140, 95% CI: 0.057–0.249) were found to significantly mediate the relationship. The direct effect (effect size = 0.866, 95% CI: 0.574–1.158) and total effect (effect size = 1.142, 95% CI: 0.848–1.435) of SPPB on MoCA were both significant.

**Conclusion:**

Higher physical function scores in older adults with cognitive impairment were associated with higher cognitive function scores. Left frontal theta and right temporal alpha2, as key observed indicators, may mediate the relationship between physical function and cognitive function. It is suggested to implement personalized exercise interventions based on the specific physical function of older adults, which may delay the occurrence and progression of cognitive impairment in older adults with cognitive impairment.

The older population in China exceeded 250 million by the end of 2020, and it is projected that by 2055, the proportion of the older population will reach 35.6% [1]. As the aging population continues to grow, the prevention or delay of cognitive decline has become an urgent issue. Cognitive function refers to the ability to select, process, store, and retrieve information, as well as apply this information to guide behavior [2]. Cognitive impairment refers to varying degrees of damage to cognitive function caused by various reasons, with an incidence rate that generally reaches 33.59% and increases with age [3]. Mild cognitive impairment (MCI) is considered a transitional stage between normal aging of the brain and dementia, and it has become a significant global public health concern [4].

Cognitive function, as a brain function, inevitably undergoes changes based on the morphological or functional changes in the brain. Electroencephalography (EEG) is an external reflection of the electrical activity of brain neurons, capable of indicating the physiological and pathological states of the brain. As a non-invasive neurophysiological detection method, EEG has advantages such as simplicity, convenience, non-invasiveness, high temporal resolution, and good spatial distribution [5, 6]. EEG can be used as an auxiliary diagnostic tool for cognitive impairment, reflecting not only pathological brain function abnormalities but also abnormalities before reaching pathological diagnostic criteria [7]. Studies have found that EEG signals in older adults with cognitive impairment exhibit specific characteristics, such as a basic rhythm slowdown, manifested by an increase in low-frequency band (delta, theta) power and a decrease in high-frequency band (alpha, beta) power [8, 9]. Slowing of alpha power may be an early sensitive indicator of the brain transitioning from normal physiological function to aging or its pathological state [10], while increased theta power may have a good predictive effect on cognitive decline [11].

There is a close relationship between physical activity and cognitive function [12]. EEG signals in individuals with cognitive impairment exhibit certain specificities, and physical activity may induce rhythmic changes in these specific indicators. Improved physical function is an external manifestation of the benefits of exercise and serves as a protective factor for cognitive function in older adults. Control of both physical and cognitive functions involves shared neural networks in the brain, and damage to the central nervous system can lead to a decline in motor function (e.g., decreased physical function) and cognitive impairment [13–15]. Physical activity promotes vascular health and increases cerebral blood flow, thereby enhancing brain plasticity by supplying adequate oxygen and nutrients to the brain [16]. Current EEG research has found that physical activity induces changes in EEG rhythms, thereby affecting cognitive function [17–19]. In individuals with cognitive impairment, acute exercise effects include decreased delta and theta power and increased beta1 power [18, 20]; long-term exercise appears to result in decreased delta [18, 20, 21] and theta rhythm power [19, 21], and increased alpha and beta rhythm power [18, 20, 22].

Literature review suggests that there is a correlation between physical function, cognitive function, and EEG indicators. Can physical function positively influence cognitive function? How do EEG indicators mediate the relationship between the two? Based on this, this study adopts a cross-sectional research design to explore the correlation between physical function, cognitive function, and EEG indicators in older adults with cognitive impairment, while clarifying the mediating role of EEG-specific indicators in the relationship between physical function and cognitive function. By monitoring changes in EEG-specific indicators in patients with cognitive impairment and regularly assessing improvements in their physical and cognitive function, this study aims to provide evidence for the development of personalized exercise programs in clinical settings. The goal is to delay the occurrence and progression of dementia in older adults with cognitive impairment.

## Research methodology

### Research design

**Fig. 1:**
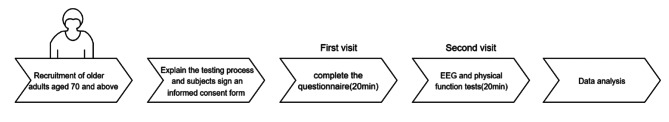
Study design procedure chart

A health education lecture was conducted in a community in Shanghai, China, aimed at recruiting older adults aged 70 and above with cognitive impairment on a voluntary basis. Research personnel explained the testing procedures to the participants, and all participants were required to sign informed consent forms (For uneducated subjects, informed consent forms are signed by the individual and their guardian). Participants were required to visit the laboratory twice. During the first visit, they completed a basic information questionnaire and the Montreal Cognitive Assessment (MoCA) Scale. Older adults who met the inclusion and exclusion criteria for cognitive impairment were required to make a second visit to the laboratory for resting-state EEG testing and physical function assessment. Participants were instructed not to engage in vigorous physical activity and refrain from consuming caffeinated or alcoholic beverages within 24 h before the tests. Please refer to the study design procedure chart (Fig. 1) for details. This study adhered to the latest version of the Helsinki Declaration ethical requirements and was approved by the Ethics Committee of Shanghai University of Sport (Approval No. 102772020RT060).

### Participants

**Fig. 2:**
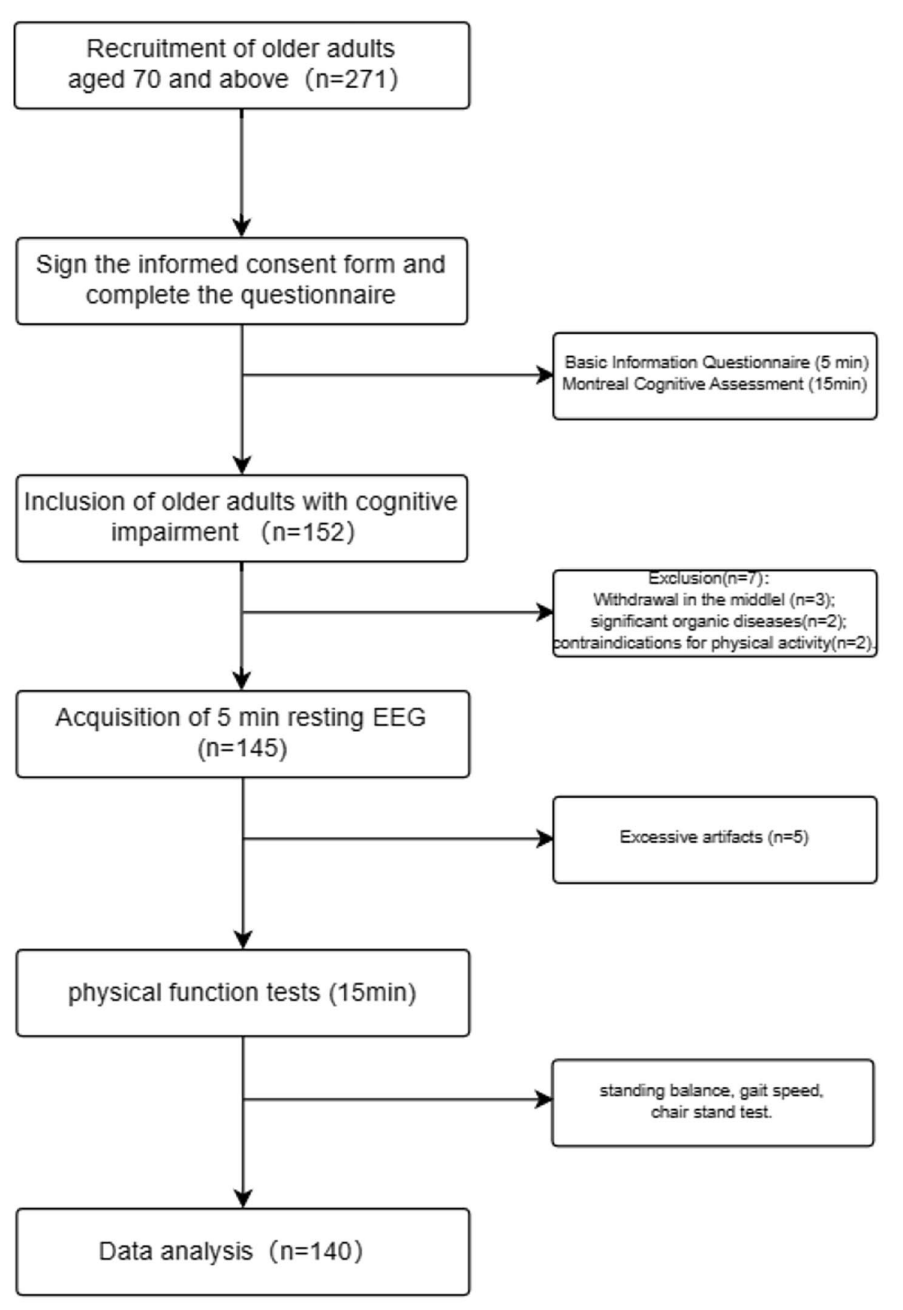
Participant flow chart

Inclusion Criteria: (1) Aged 70 years and above. (2) Montreal Cognitive Assessment score < 26. (3) Absence of major organic diseases. (4) No contraindications to exercise. (5) Ability to communicate verbally, cooperate to complete the survey, and acceptable visual, auditory, and mental conditions. (6) Willingness to sign informed consent. Exclusion Criteria: (1) Severe cardiovascular diseases. (2) Severe mental illnesses. (3) Severe musculoskeletal disorders. (4) Unwillingness to participate in the survey. (5) Incomplete questionnaire response. (6) Excessive artifacts in the EEG recordings. A total of 271 older adults aged 70 and above were recruited. Research personnel screened the older adults using the Montreal Cognitive Assessment Scale, and 152 were found to have cognitive impairment, with a detection rate of 56.09%. After excluding participants who did not meet the inclusion and exclusion criteria, a total of 140 older adults with cognitive impairment were included in the data analysis. Please refer to the participant flow chart (Fig. 2) for details.

### Testing tools

#### Basic information questionnaire

The basic information questionnaire includes the following details: name, age, gender, height, weight, marital status, past occupation, education level, presence of exercise contraindications, medical history, and medication usage.

#### Montreal cognitive assessment

Widely used for assessing cognitive function in the older adults, MoCA consists of 8 aspects: visuospatial/executive function, naming, memory, attention, language fluency, abstraction, delayed recall, and orientation. The total score is 30 points, with higher scores indicating better cognitive function [23]. MoCA scores above or equal to 26 are considered normal, scores between 18 and 26 indicate mild cognitive impairment, scores between 10 and 17 indicate moderate impairment, and scores below 10 indicate severe impairment. To account for the impact of education duration, 1 point is added to the total score for education levels of 12 years or less; if the total score exceeds 30 after adding, no additional points are given. The retest reliability of MoCA used in this study is 0.857 [23].

#### Short physical performance battery (SPPB)

The physical function test utilized the Short Physical Performance Battery, developed by American scholar Guralnik and colleagues in 1994 [24]. This assessment battery includes three components: the standing balance test, gait speed test (over a 4-meter distance), and chair stand test (five repetitions), which collectively reflect an older adult’s individual standing balance, gait speed, and lower limb muscle strength [25]. The SPPB total score is obtained by summing the scores from the three tests, with a scale range of 0 to 12 points. Scores from 0 to 6 indicate frailty, 7 to 9 suggest pre-frailty, and 10 to 12 indicate non-frailty.

#### EEG signal acquisition and processing

The testing environment was an indoor setting with appropriate soundproofing, ventilation, and lighting, and maintained moderate temperature and humidity levels, with no electromagnetic interference. Prior to testing, participants washed their hair and allowed it to air dry, then assumed a seated position and adjusted to a comfortable posture. Test instructions were given to the participants to close their eyes and remain relaxed, quiet, and awake, avoiding blinking, teeth clenching, swallowing, or body movement. EEG data were recorded using the Shanghai Nuocheng Electric Co., Ltd. EEG recorder (NCERP-190,012), with 16 electrodes placed according to the international 10/20 system: Fp1 (left frontal), Fp2 (right frontal), F3 (left frontal), F4 (right frontal), C3 (left central), C4 (right central), P3 (left parietal), P4 (right parietal), O1 (left occipital), O2 (right occipital), F7 (left frontotemporal), F8 (right frontotemporal), T3 (left temporal), T4 (right temporal), T5 (left posterior temporal), and T6 (right posterior temporal), with the ground electrode placed in the midline of the forehead, and reference electrodes placed on both mastoids (A1 and A2). The instrument was equipped with preamplifiers, with a sampling frequency set to 500 Hz, high-pass filtered at 0.3 Hz, low-pass filtered at 30 Hz, and electrode-skin impedance maintained below 5 kΩ. EEG data were collected during a 5-minute resting period for each participant.

Resting-state EEG data were processed using EEGLAB in MATLAB, following the main procedures outlined below: (1) Data Import: EEG data were imported into EEGLAB for further analysis. (2) Electrode Localization: The analysis focused on the 16 electrodes specified earlier, with the reference electrodes A1 and A2 selected for re-referencing. (3) Artifact Removal: Independent Component Analysis algorithm was employed to remove artifacts from the EEG data. (4) Frequency Band Division: The EEG spectrum was divided into six frequency bands: delta (1–4 Hz), theta (4–8 Hz), alpha1 (8–10.5 Hz), alpha2 (10.5–13 Hz), beta1 (13–20 Hz), and beta2 (20–30 Hz). (5) Fourier Transform: Fourier transform was applied to calculate the spectrum or power spectrum of the EEG data. (6) Power Calculation: For each electrode, the power values within each frequency band were computed using the method of averaging.

### Statistical analysis

We conducted statistical analysis using SPSS 26.0 software. Continuous data are presented as mean ± standard deviation, while categorical data are described by counts (n). The relationship between physical function and cognitive function was examined using Pearson correlation analysis, one-way analysis of variance, and post-hoc multiple comparisons with Bonferroni correction. Pearson correlation analysis was used to investigate the relationship between cognitive function and EEG indicators. After Bonferroni correction, the significance level was set at 0.00052, where *p* < 0.00052 indicates statistical significance. Linear regression analysis was employed to explore the extent to which EEG indicators explain cognitive function. Pearson correlation analysis was utilized to examine the relationship between physical function and EEG-specific indicators. After Bonferroni correction, the significance level was set at 0.01667, where *p* < 0.01667 indicates statistical significance. We utilized Model 4 in PROCESS to analyze the mediating effect of EEG-specific indicators between physical function and cognitive function. Path analysis parameter estimation was conducted using the Bootstrap method with 5000 resamples. A 95% confidence interval (CI) excluding zero was considered to indicate statistical significance for the mediating effect. All statistical inferences were two-tailed tests with a significance level (α) set at 0.05, representing statistical significance (without the need for Bonferroni correction in data analysis).

## Results

### Basic information of older adults with cognitive impairment

140 older adults with cognitive impairment participated in the analysis (Table 1), with a mean age of 72.51 ± 7.76 years and a mean BMI of 24.06 ± 3.23. There were 80 male participants and 60 female participants.

**Table 1:** Basic Information of Older Adults with Cognitive Impairment (*N* = 140)

Variables	Older adults with cognitive impairment
Age, mean ± SD	72.51 ± 7.76
BMI kg/m^2^, mean ± SD	24.06 ± 3.23
Sex	
Male	80
Female	60
Marital Status	
Married	112
Unmarried, Divorced or Widowed	28
Educational level	
Uneducated	33
Elementary school	70
Middle school	30
High school and above	7

### Relationship between physical function and cognitive function

**Fig. 3:**
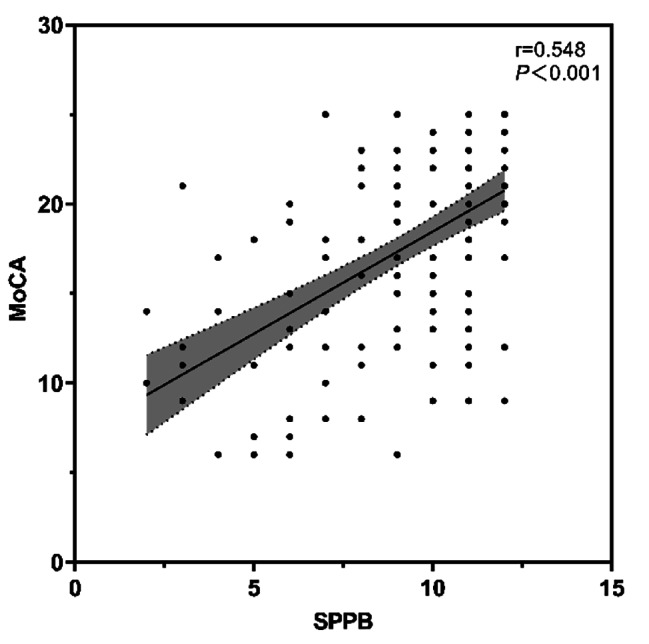
Relationship between SPPB Scores and MoCA Scores (*N* = 140)

We examined the relationship between physical function and cognitive function using Pearson correlation coefficient (Fig. 3). The SPPB score was significantly positively correlated with the MoCA score (*r* = 0.548, *p* < 0.001). Among older adults without physical frailty (*n* = 75), the MoCA score was 19.65 ± 4.54; among those in the pre-frail stage (*n* = 37), the MoCA score was 16.86 ± 5.06; and among physically frail older adults (*n* = 28), the MoCA score was 12.36 ± 5.18. Differences in cognitive function among older adults with different physical function statuses were statistically significant (F = 23.837, *p* < 0.001). Post-hoc multiple comparisons revealed that the differences between older adults without physical frailty and those in the pre-frail and frail stages were statistically significant (*p* < 0.05), and the difference between those in the pre-frail and frail stages was also statistically significant (*p* < 0.001).

### Selection of EEG-Specific indicators for cognitive function

We examined the relationship between EEG indicators and cognitive function using Pearson correlation coefficients. As shown in Table 2, a total of 31 EEG indicators were significantly correlated with MoCA scores (all *p* < 0.00052).

**Table 2:** Correlation Coefficients between EEG Indicators and MoCA Scores (*N* = 140)

	delta	theta	alpha1	alpha2	beta1	beta2
FP1	-0.274	-0.380^*^	0.387^*^	0.304^*^	0.047	-0.182
FP2	0.039	-0.299^*^	0.393^*^	0.273	0.089	-0.193
F3	-0.059	-0.123	0.399^*^	0.284	0.059	-0.150
F4	0.051	-0.061	0.414^*^	0.323^*^	0.160	-0.026
C3	0.053	-0.072	0.404^*^	0.342^*^	0.162	-0.117
C4	0.056	-0.096	0.404^*^	0.345^*^	0.212	-0.008
P3	0.082	-0.062	0.406^*^	0.344^*^	0.180	0.022
P4	0.050	-0.070	0.393^*^	0.365^*^	0.249	0.057
O1	0.042	-0.105	0.400^*^	0.346^*^	0.088	-0.123
O2	0.081	-0.134	0.390^*^	0.351^*^	0.184	-0.043
F7	0.092	-0.065	0.412^*^	0.365^*^	0.196	0.018
F8	0.071	-0.059	0.400^*^	0.366^*^	0.244	0.050
T3	-0.016	-0.164	0.302^*^	0.187	-0.090	-0.251
T4	0.179	0.126	0.365^*^	0.321^*^	0.090	-0.082
T5	0.041	-0.096	0.382^*^	0.331^*^	0.083	-0.172
T6	0.077	-0.131	0.388^*^	0.349^*^	0.185	-0.069

Note: ^*^ indicates *p* < 0.00052, indicating statistical significance

To examine the explanatory power of EEG indicators on cognitive function, multiple linear regression analysis (stepwise method) was conducted with MoCA score as the dependent variable and all EEG indicators as independent variables. The results (Table 3) showed that the regression model passed the significance test (F = 18.141, *p* < 0.001, R^2^ = 0.526). F4 alpha1, FP1 theta, T4 alpha2, P3 delta, T6 theta, F3 theta, FP1 beta2, and F4 delta were identified as influencing factors of MoCA scores in older adults, explaining 52.6% of the variance in cognitive function. The tolerance values for each independent variable were all > 0.1, and the variance inflation factor (VIF) values were all < 5, indicating that multicollinearity was effectively controlled for in this study.

**Table 3:** Multiple Linear Regression Analysis of EEG Indicators Explaining Cognitive Function (*N* = 140)

Independent variables	B	95%CI		Beta	Coefficient significance test		SE	Collinearity diagnostics	
		Lower limit	Upper limit		*t*-value	*p* value		Tolerance	VIF
F4 alpha1	0.263	0.128	0.398	0.256	9.866	<0.001	0.068	0.821	1.218
FP1 theta	-1.105	-1.469	-0.741	-0.681	3.850	<0.001	0.184	0.282	3.551
T4 alpha2	0.460	0.222	0.698	0.263	-6.005	<0.001	0.120	0.767	1.304
P3 delta	1.666	0.731	2.600	0.369	3.822	0.001	0.473	0.330	3.031
T6 theta	-0.369	-0.588	-0.149	-0.391	3.525	0.001	0.111	0.261	3.832
F3 theta	0.669	0.248	1.089	0.386	-3.321	0.002	0.213	0.241	4.151
FP1 beta2	-0.125	-0.222	-0.029	-0.179	3.144	0.012	0.049	0.737	1.356
F4 delta	2.300	0.008	4.592	0.190	-2.560	0.049	1.158	0.397	2.522
Model summary: F = 18.141, *p*<0.001, *R* = 0.725, R^2^=0.526, Adjusted R^2^=0.497									

Note: B indicates unstandardized coefficients. Beta indicates standardized coefficients. SE indicates Standard Error

The above results indicate that among the EEG indicators, F4 alpha1, FP1 theta, and T4 alpha2 are significantly correlated with MoCA scores and are retained as predictor variables in the regression model. These three variables represent EEG-specific indicators of cognitive function.

### Physical function and EEG specific indicators

**Fig. 4:**
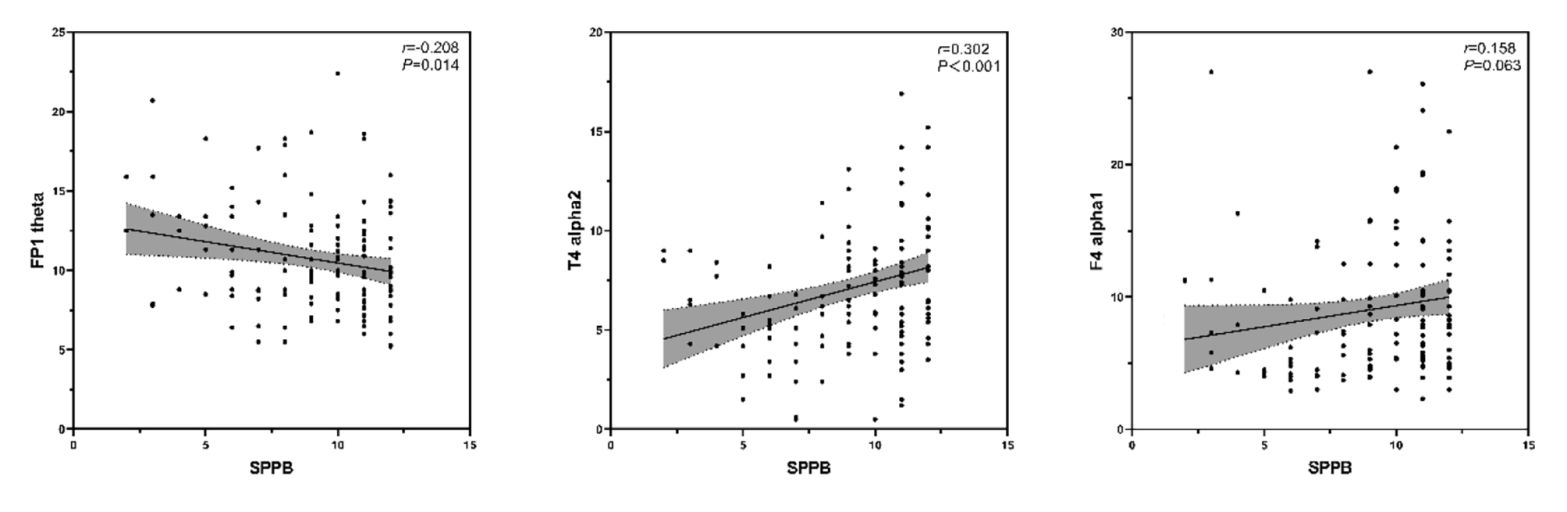
Relationship between SPPB Scores and EEG-Specific Indicators (*N* = 140)

We examined the relationship between physical function and EEG-specific indicators in older adults using Pearson correlation coefficients. As shown in Fig. 4, SPPB scores were significantly negatively correlated with FP1 theta (*r* = -0.208) and significantly positively correlated with T4 alpha2 (*r* = 0.302), with both *p*-values < 0.01667. SPPB scores were positively correlated with F4 alpha1 (*r* = 0.158), but this correlation did not reach statistical significance (*p* = 0.063).

### Pathway of EEG-Specific indicators mediating the relationship between physical function and cognitive function

Based on the interrelationships among physical function, EEG-specific indicators, and cognitive function, we utilized SPPB scores as the independent variable and MoCA scores as the dependent variable to analyze the mediating effects of FP1 theta and T4 alpha2 using Model 4 in PROCESS. The results (Table 4) showed that SPPB negatively predicted FP1 theta (Beta = -0.208, *p* < 0.05) and positively predicted T4 alpha2 (Beta = 0.302, *p* < 0.001), which in turn positively predicted MoCA scores (Beta = 0.416, *p* < 0.001). FP1 theta negatively predicted MoCA scores (Beta = -0.313, *p* < 0.001), while T4 alpha2 positively predicted MoCA scores (Beta = 0.223, *p* < 0.01).

**Table 4:** Regression Analysis of Variable Relationships in the Mediation Model (*N* = 140)

variables	Model 1		Model 2		Model 3	
	Beta	*t*	Beta	*t*	Beta	*t*
SPPB	-0.208	-2.501^*^	0.302	3.723^***^	0.416	5.868^***^
FP1 theta					-0.313	-4.612^***^
T4 alpha2					0.223	3.206^**^
R^2^	0.043		0.091		0.418	
F	6.255^*^		13.859^***^		32.559^***^	

Note: Model 1 indicates SPPB predicts FP1 theta; Model 2 indicates SPPB predicts T4 alpha2; Model 3 indicates SPPB, FP1 theta, and T4 alpha2 jointly predict MoCA. Beta indicates standardized coefficients. ^*^ indicates *p* < 0.05; ^**^ indicates *p* < 0.01; ^***^ indicates *p* < 0.001

**Fig. 5:**
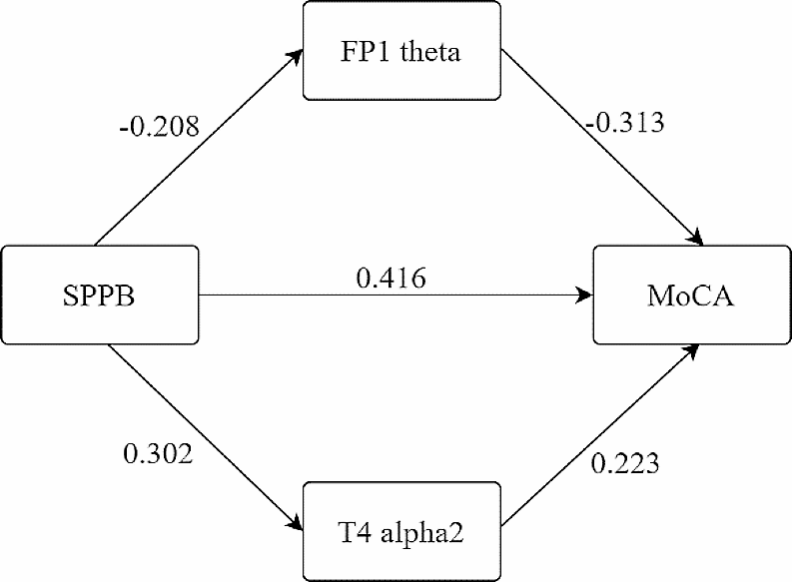
Structural Model of the Relationship between Physical Function, EEG-Specific Indicators, and Cognitive Function (*N* = 140) Note: Path values are standardized coefficients

After further testing using bias-corrected bootstrap method, the total effect of SPPB on MoCA was found to be 1.142 (95% CI: 0.848–1.435). The direct effect of SPPB on MoCA was 0.866 (95% CI: 0.574–1.158). The mediating effect of FP1 theta was 0.136 (95% CI: 0.025–0.251), and the mediating effect of T4 alpha2 was 0.140 (95% CI: 0.057–0.249). Both the total effect and direct effect of SPPB on MoCA were significant, as well as the mediating effects of FP1 theta and T4 alpha2. Please refer to Table 5; Fig. 5 for details.

**Table 5:** Bootstrap Analysis for Significance Testing of Mediation Effects (*N* = 140)

Item	effect size	Boot SE	Bootstrap 95%CI	
			Lower limit	Upper limit
Total Effect	1.142	0.148	0.848	1.435
Direct Effect	0.866	0.148	0.574	1.158
FP1 theta	0.136	0.057	0.025	0.251
T4 alpha2	0.140	0.049	0.057	0.249

## Discussion

The results of this study demonstrate that older adults with cognitive impairment tend to have higher physical function scores, which are positively correlated with cognitive function scores. This finding aligns with previous research findings [26]. There is a significant positive correlation between physical function and cognitive function [27, 28], with muscle strength, walking ability, balance, and aerobic endurance closely related to cognitive function [26, 29, 30]. Increased physical activity is associated with improved physical function, which can increase cerebral blood flow, providing sufficient oxygen to the brain [31]. This supports the nourishment and energy metabolism of brain cells, promotes neuron survival, and synapse generation [32]. Physical activity not only enhances the volume of brain structures such as the hippocampus and cerebellum but also influences the level of brain activation and functional connectivity between brain regions, thereby improving cognitive function [32].

The left frontal theta wave, located at the front of the brain, may mediate the relationship between physical and cognitive functions in older adults with cognitive impairment. The left frontal area, located at the front of the brain, is associated with advanced cognitive functions, decision-making, problem-solving, and personality traits. It plays a role in executive functions, emotional regulation, and modulation of social behavior. The theta wave typically appears during shallow sleep, relaxation, or periods of inattentiveness in awake states. In adults, its presence may be linked to decreased consciousness or cognitive issues. The study revealed that changes in brain function and structure can lead to abnormal brain waves [33]. In older adults with cognitive impairment, there is degeneration in brain tissues, significant loss of neurons in the frontal lobe cortex, and a reduction in the number of synaptic connections [34]. This results in decreased function of the frontal lobe cortex, manifested by slower brain wave frequencies and the emergence of theta waves [34, 35]. The severity of cognitive impairment is positively correlated with increased theta activity, and an increase in theta waves in EEG serves as a good predictor of cognitive decline [36]. Higher levels of physical activity can increase cerebral blood flow, enhance oxygen supply, and promote neural nutrition [31], contributing to improved function of the frontal lobe cortex. As the activation level of the frontal lobe cortex increases, theta wave power decreases, leading to improved cognitive function. A decrease in theta wave power was observed after both single and prolonged exercise [17].

This study also identified that the right middle temporal alpha2 waves may mediate the relationship between physical and cognitive functions in older adults with cognitive impairment. The temporal region is associated with auditory and language processing, emotional regulation, and social behavior. The right middle temporal lobe may be involved in auditory and language tasks. Alpha2 waves are generally associated with higher alertness and cognitive activity, serving as an indicator of a relaxed yet alert state. The research found noticeable atrophy in the hippocampus, amygdala, and temporal lobe of older adults with cognitive impairment [37]. Decreased glucose metabolism and blood perfusion were observed in regions such as the temporal parietal lobule and cingulate gyrus [38]. This led to impaired function in the temporal region, manifested by a significant decrease in alpha power. Alpha waves are a crucial indicator of cognitive function, and alpha slowing is considered an early sensitive marker of the brain transitioning from normal physiological function to aging or pathological states [10]. Individuals with cognitive impairment exhibit a reduction in Alpha2 power in the temporal region [36, 39]. Increased physical activity can stimulate the release of brain-derived neurotrophic factor, enhancing the activity of glutamatergic neurons in the hippocampus, promoting long-term memory storage, and facilitating the growth and restructuring of dendrites in response to changes in neuronal activity [40, 41]. It can also increase cerebral blood flow, elevate activation in the temporal region, increase alpha2 wave power, and thus improve language function, auditory perception, and long-term memory. An increase in Alpha2 wave power was observed after both single and prolonged exercise [17].

This study further elucidates the neurophysiological mechanisms underlying cognitive decline in older adults. Left frontal theta and right middle temporal alpha2 can serve as key observational indicators for identifying and preventing cognitive decline in older adults. Clinically, by monitoring changes in EEG-specific indicators of cognitive impairment patients and regularly assessing improvements in patients’ physical and cognitive function, exercise intervention plans can be adjusted in a timely manner to ensure older adults obtain the optimal effects of exercise intervention. This may delay the progression of cognitive impairment in older adults to dementia.

However, this study has several limitations: it is a cross-sectional study, and further longitudinal research is needed to confirm the causal pathways; the study did not explore whether other moderating variables affect EEG indicators when mediating effects, and future research could investigate the effects of moderating variables; the study included non-clinically diagnosed participants, limiting the generalizability of the results.

## Conclusion

Higher physical function scores in older adults with cognitive impairment were associated with higher cognitive function scores. Left frontal theta and right temporal alpha2, as key observed indicators, may mediate the relationship between physical function and cognitive function. It is suggested to implement personalized exercise interventions based on the specific physical function of older adults, which may delay the occurrence and progression of cognitive impairment in older adults with cognitive impairment.

## Data Availability

No datasets were generated or analysed during the current study.

## Declarations

### Data and materials sharing

Materials and analysis code for this study are available by emailing the corresponding author.
